# The Association between the Consumption of Fish/Shellfish and the Risk of Osteoporosis in Men and Postmenopausal Women Aged 50 Years or Older

**DOI:** 10.3390/nu8030113

**Published:** 2016-02-25

**Authors:** Eunjin Choi, Youngsoon Park

**Affiliations:** Department of Food and Nutrition, Hanyang University, 222 Wangsimni-ro, Seongdong-gu, Seoul 04763, Korea; eunjin06@hanyang.ac.kr

**Keywords:** fish and shellfish, *n*-3 polyunsaturated fatty acids, bone mineral density, osteoporosis, elderly population, KNHANES, NHANES

## Abstract

Fish rich in *n*-3 polyunsaturated fatty acids have been suggested to have a favorable effect on bone health, but previous epidemiologic studies have shown inconsistent results. The purpose of the present study was to investigate the hypothesis that the consumption of fish and shellfish is positively associated with bone mass and negatively associated with the risk of osteoporosis in Koreans and Americans. Men and postmenopausal women ≥50 years old from the Korean National Health and Nutrition Examination Survey 2008–2011 (*n* = 7154) and the National Health and Nutrition Examination Survey 2007–2010 (*n* = 2658) were included. There was a positive correlation between the consumption of fish and shellfish and bone mineral density (BMD) of the total femur, femoral neck, and lumbar spine in Koreans. Consistently, multivariate logistic regression analysis showed a significant association between intake of fish and shellfish and the risk of osteoporosis in Koreans but not in Americans. Consumption of fish and shellfish was 4–5 times higher in Koreans than Americans in the present study. In conclusion, intake of fish and shellfish was associated with BMD and the risk of osteoporosis in Koreans but not in Americans, suggesting that a minimum intake level of fish and shellfish might be recommended to protect against bone loss and osteoporosis.

## 1. Introduction

Osteoporosis is characterized by low bone mass with consequent increases in susceptibility to fracture, and it is considered to be a major public health problem affecting particularly postmenopausal women and elderly people in general [1]. Several nutritional and dietary factors have been identified as being important for maintaining bone health, preventing loss of bone mineral density (BMD), and reducing the risk of osteoporosis in old age [2]. Particularly, the roles of calcium and vitamin D are well established in bone health [2,3]. Previously, we reported that the blood level of *n*-3 polyunsaturated fatty acids was associated with bone health in Korean menopausal women [4].

Intake of fish rich in *n*-3 PUFAs, such as eicosapentaenoic acid (EPA; 20:5*n*3) and docosahexaenoic acid (DHA; 22:6*n*3), can favorably modulate bone health [5]. *N*-3 PUFAs have been suggested to suppress the production of inflammatory cytokines [6], enhance calcium absorption [7], and reduce urinary calcium excretion [8]. Epidemiologic studies have reported that intake of fish and *n*-3 PUFAs was significantly associated with BMD [9], fracture [10], and the risk of osteoporosis [11] in postmenopausal women or elderly men in an Asian population. However, in Western populations, the consumption of *n*-3 PUFAs or fish was not found to be associated with BMD [12] or fracture [13,14,15,16,17] in elderly men or women, except for one study showing that the consumption of *n*-3 PUFAs and dark fish was positively associated with BMD in elderly men [18].

Previous studies have focused on the association of fish intake and bone health, but shellfish also contain *n*-3 PUFAs [19]. Shellfish intake had a significant protective effect on the risk of hip fracture in elderly Chinese [10], and the consumption of fish and shellfish was positively associated with BMD in Chinese women [20]. American adults have been reported to consume shellfish and fish at a 4:6 ratio [21], and Korean adults consume shellfish and fish at a 3:7 ratio [22]. Consumption of fish and shellfish, rather than the consumption of fish alone, could be more closely related to bone health, but there has been no study comparing the association between fish and shellfish consumption and the risk of osteoporosis. Therefore, the purpose of the present study was to investigate the hypothesis that the consumption of fish and shellfish is positively associated with bone mass and negatively associated with the risk of osteoporosis in Korean and American men and postmenopausal women ≥50 years old.

## 2. Materials and Methods

### 2.1. Participants

This study was based on data from the Korean National Health and Nutrition Examination Survey (KNHANES) 2008–2011 and the National Health and Nutrition Examination Survey (NHANES) 2007–2010. The surveys were completed on a stratified, multistage, clustered national probability sample of non-institutionalized civilians. Data were included from men and postmenopausal women ≥50 years old who had undergone a checkup for osteoporosis. Participants were excluded if they used hormone replacement therapy or were missing data on dietary intake or BMD. Participants were also excluded if their daily energy intake was lower than the 1st percentile or higher than the 99th percentile. Thus, 7154 participants from the KNHANES and 2658 participants from the NHANES were included after excluding 30,599 from the KNHANES and 17,357 from the NHANES. The study protocol was approved by the Institutional Review Boards of Hanyang University (HYI-15-069).

### 2.2. Dietary Intake

Participants were interviewed by a trained dietitian using 24-h recall and a food frequency questionnaire (FFQ). Total energy and intake of protein, sodium, and calcium were determined using 24-h recall and were calculated by multiplying the food code-specific nutrient concentration data by the corresponding weight of each reported food. All reported items were coded using the US Department of Agriculture Food and Nutrient Database for the NHANES 2007–2008 [23] and 2009–2010 [24] or Korea Food Composition Table for the KNHANES [25], which provided nutritional content based on standardized recipes. The frequency of consuming fish and shellfish without portion size was collected by FFQ. Participants in the KNHANES were asked if they had consumed any of 5 species of fish and shellfish during the previous 12 months, whereas those in the NHANES were asked if they had consumed any of 31 species of fish and shellfish over the past 30 days.

### 2.3. Bone Mass

BMDs of total femur, femoral neck, and lumbar spine were measured using dual-energy X-ray absorptiometry (DXA; Discovery-W, Hologic, Inc., Waltham, MA, USA for the KNHANES and QDR 4500A, Hologic, Bedford, MA, USA for NHAENS). The prevalence of osteoporosis was defined by diagnosis by a physician in the KNHANES and the NHANES.

### 2.4. Risk Factors

The risk factors of osteoporosis included in the analysis were body mass index (BMI), smoking, alcohol, exercise, family history, and medical history, which are factors suggested by the International Osteoporosis Foundation (IOF), the National Osteoporosis Foundation (NOF), and the Fracture Risk Assessment Tool (FRAX) from the World Health Organization (WHO) [26,27,28]. BMI was calculated using measured height and weight; smoking state, alcohol intake, regular exercise, family history of osteoporosis or fracture, and medical history of rheumatoid arthritis, osteoporosis, and thyroid disease were obtained by interview in the KNHANES and the NHANES.

### 2.5. Statistical Analyses

All statistical analyses were performed using SAS software (version 9.3; SAS Institute, Cary, NC, USA). Survey weights were used to generate nationally representative estimates for both populations and were adjusted for the complex sample design of the KNHANES and the NHANES. *p*-values < 0.05 (two-sided) were considered statistically significant. A two-step modeling strategy was used to adjust for potential confounding variables and to improve the precision in estimating the effects of consuming fish and shellfish on BMD. Variables that were associated with BMD with *p*-value ≤ 0.25 in bivariate analyses were included in the full model. A backward-elimination approach was used to exclude potential covariates from the final model. Covariates were retained in the final model if the *p*-value was ≤0.01. Data were presented as the mean ± standard deviation (SD) for continuous variables or as a percentage for categorical variables. Differences were analyzed using the Chi-square test for categorical variables and Student’s *t*-test for continuous variables. The relationship between the consumption of fish and shellfish, and BMD was analyzed using a partial Pearson’s correlation analysis after adjusting for BMI, smoking state, regular exercise, intake of calcium, family history of osteoporosis or fracture, and medical history of rheumatoid arthritis. The multiple comparisons were calculated by the analysis of covariance (ANCOVA) with a Bonferroni’s *post hoc* test. In the ANCOVA models, adjustments were made for BMI, smoking state, regular exercise, intake of calcium, family history of osteoporosis or fracture, and medical history of rheumatoid arthritis. Odds ratios (ORs) and 95% confidence intervals (CIs) for the risk of osteoporosis according to the consumption of fish and shellfish were analyzed by multivariate logistic regression models. Model 1 was not adjusted; Model 2 was adjusted for BMI, regular exercise, and intake of calcium; Model 3 was adjusted for BMI, regular exercise, intake of calcium, smoking state, family history of osteoporosis or fracture, and medical history of rheumatoid arthritis. The lowest quintile of fish and shellfish consumption was considered as a reference, and the likelihood ratio test was used for detecting trends.

## 3. Results

### 3.1. Characteristics of Participants

Korean men and postmenopausal women had lower BMI; BMDs of total femur, femoral neck, and lumbar spine; lower intakes of protein, calcium, and alcohol; and frequency of medical history of thyroid disease. However, they had greater intakes of fish, shellfish, and sodium, and a higher exercise rate than American men and postmenopausal women (Table 1). Korean men and American postmenopausal women had higher smoking rates than American men and Korean postmenopausal women, respectively. Korean men had less frequent medical history of rheumatoid arthritis than American men, and Korean postmenopausal women were older and had had less frequent family history of osteoporosis or fracture than did American postmenopausal women. The prevalence of osteoporosis was not different between Korean and American men and postmenopausal women.

### 3.2. Association between Consumption of Fish and Shellfish and BMD

**Figure 1:**
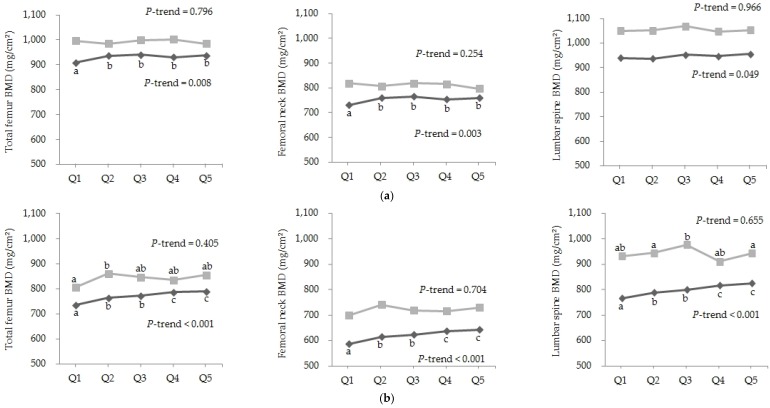
Association between bone mineral density (BMD) and quintile (Q) of the consumption of fish and shellfish (times/month) in men ≥50 years old (**a**) and postmenopausal women ≥50 years old (**b**) from the Korean National Health and Nutrition Examination Survey (KNHANES, 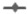) and National Health and Nutrition Examination Survey (NHANES, 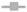). Q1 (4.2), Q2 (10.9), Q3 (19.3), Q4 (30.4), Q5 (49.7) in men of KNHANES; Q1 (0), Q2 (1.5), Q3 (3.5), Q4 (5.8), Q5 (12.2) in men of NHANES; Q1 (3.1), Q2 (8.5), Q3 (16.6), Q4 (28.3), Q5 (49.3) in women of KNHANES; and Q1 (0), Q2 (1.5), Q3 (3.6), Q4 (6.0), Q5 (11.9) in women of NHANES. Values with different letters are significantly different with a *p*-value < 0.05 (ANCOVA with Bonferroni’s *post hoc* test).

There was a positive correlation between the consumption of fish and shellfish and BMD of total femur, femoral neck, and lumbar spine in Korean men and postmenopausal women after adjusting for confounding variables (Table 2). However, there was no significant correlation between the consumption of fish and shellfish and BMD in American men or postmenopausal women. Consistently, BMD of total femur, femoral neck, and lumbar spine was significantly and positively associated with each corresponding quintile of consumption of fish and shellfish in Korean men and postmenopausal women (Figure 1). However, there was no significant association between the consumption of fish and shellfish and BMD in American men or postmenopausal women. In Americans, BMD of total femur, femoral neck, and lumbar spine was not significantly associated with the consumption of fish and shellfish. In particular, there were significant dose-dependent associations between intake of fish and shellfish and BMD of total femur, femoral neck, and lumbar spine in Korean postmenopausal women.

### 3.3. Association between Consumption of Fish and Shellfish and Risk of Osteoporosis

Multivariate logistic regression analysis showed a significant association between intake of fish and shellfish and the risk of osteoporosis in Korean men and postmenopausal women before and after adjustment for confounding variables (Table 3). In American postmenopausal women, the consumption of fish and shellfish was associated with a lower risk of osteoporosis in Model 1 using crude data and in Model 2, where the data were adjusted for BMI, regular exercise, and intake of calcium. However, this association was not seen after adjustment for BMI, regular exercise, intake of calcium, smoking state, family history of osteoporosis or fracture, and medical history of rheumatoid arthritis in Model 3. In American men, there was no association between the consumption of fish and shellfish and the risk of osteoporosis in all models.

## 4. Discussion

The present study showed that the consumption of fish and shellfish was positively associated with bone mass and negatively associated with the risk of osteoporosis in Korean men and postmenopausal women ≥50 years old but not in Americans. Previous epidemiologic studies have consistently reported the beneficial effect of seafood [20] and fish [11,29] on BMD in Asian populations. However, the majority of studies conducted in Americans reported no association between fish intake and BMD [12,18]. The Framingham Osteoporosis Study also showed that fish intake was not associated with BMD, but BMD was significantly higher in the group consuming ≥340 g/week of dark fish compared to those consuming <113 g/week [18]. Dark fish is known to contain more *n*-3 PUFAs than other fish [19].

Consistent with the present study, previous studies have reported that the effect of fish/seafood on BMD was different between Asian and American populations. The inconsistencies may be related to the heterogeneity of the populations. Another possible explanation could be the difference in fish intake between Asians and Americans. Regarding BMD, effective intake of fish/seafood was 250–833 g/week in an Asian population [11,20,29]. The KNHANES 2010 reported that the average intake of fish and shellfish was ~353 g/week in elderly Koreans [30], and the present study showed that the average intake of fish and shellfish was 5.8 times/week. On the other hand, the NHANES 2005–2010 reported that the average intake of fish and shellfish was ~158 g/week in Americans [21], and the Cardiovascular Health Study also showed that the average fish intake was 113.5 g/week in American elderly [12]. Additionally, the present study reported that the average intake of fish and shellfish was 1.3 times/week in American men and postmenopausal women ≥50 years old. Intake of fish and shellfish between quintile 1 and 2 in the KNHANES was similar to those between quintile 3 and 5 in the NHANES, which could explain the different effect of fish and shellfish on bone health. Finally, inconsistencies between Korean and American populations may be related to the cooking methods of fish and shellfish. The common method of cooking is frying in the Western population [31], while it is boiling or steaming in Korea [32]. Frying fish and shellfish decreased *n*-3 PUFA content as compared with boiling or steaming fish.

Epidemiologic studies have reported that intake of *n*-3 PUFA was positively associated with BMD in a Japanese population, whose average intake of *n*-3 PUFA was 2.7 g/day [9], but this was not the case in an American population, whose *n*-3 PUFA consumption was 0.15–0.5 g/day [12,18,33]. Nawata *et al.* [9] also showed that *n*-3 PUFA intake was positively associated with serum N-terminal propeptide of type I collagen, a biomarker of bone formation, and negatively associated with urinary type-I collagen cross-linked-*N*-telopeptide (NTx), a biomarker of bone resorption, in a Japanese population. In addition, a diet rich in α-linolenic acid (18:3n3) was found to decrease the serum concentrations of NTx and tumor necrosis factor-α (TNF-α) compared to an average American diet [34]. Reduction of TNF-α induced by *n*-3 PUFA can decrease prostaglandin E_2_ formation, which stimulates the differentiation of osteoclast and bone formation through increased production of insulin-like growth factor, a powerful growth stimulator of bone and muscle [35]. *N*-3 PUFAs have also been shown to enhance calcium absorption [7] and reduce urinary calcium excretion [8].

In addition to bone mass, the risk of osteoporosis has been shown to be negatively associated with the consumption of fish in Chinese postmenopausal women [11]. An animal study also reported that reduction of BMD was significantly lower in ovariectomized (OVX) mice fed fish oil than in OVX mice fed corn oil [5]. Additionally, treatment with EPA and/or DHA inhibited nuclear factor-kappa B, which regulates osteoclast formation in bone marrow cell-derived macrophages [5], suggesting that *n*-3 PUFAs protect against osteoporosis. The present study showed that Korean men and women consumed fish and shellfish an average of ≥49.5 times/month, and this level of consumption was associated with a lower risk of osteoporosis, while American men and women consumed fish and shellfish an average of ≤3.6 times/month, a level that was not associated with decreased risk of osteoporosis.

Consistent with studies on BMD and the risk of osteoporosis, epidemiologic studies have reported inconsistent results regarding the effects of seafood, fish, and *n*-3 PUFA on hip fracture. The risk of hip fracture was lower in Chinese men and women consuming ≥502 g/week of fish and shellfish than in those consuming <61 g/week [10]. However, there was no association between the risk of hip fracture and seafood/fish intake in Europeans, whose average consumption of fish and shellfish was 140 g/week [36], or in Americans, whose average consumption of fish was 77–224 g/week [12,13,15].

This study had a few limitations. First, because of the cross-sectional study design, it was unable to establish a cause-effect relationship between bone health and the consumption of fish and shellfish. Second, the consumption of fish and shellfish was estimated by qualitative FFQ without portion size. Third, the present study did not included the intake of calcium, vitamin D and other nutrients related to bone health in the statistical model because all of the nutrients were associated with BMD with *p*-value ≤ 0.25 in bivariate analyses. Finally, the blood level of vitamin D was not included in the study due to the lack of such data in the KNHANES. Fish, particularly dark fish, is the major dietary source of both *n*-3 PUFA and vitamin D, which have been shown to be negatively associated with the risk of osteoporosis and positively associated with BMD. Previously, Park *et al.* [37] observed that the serum level of 25-hydroxyvitamin D was positively associated with tissue level of *n*-3 PUFA, suggesting that intake of fish is not only a contributor to *n*-3 PUFA, but also to vitamin D.

## 5. Conclusions 

The present study found a positive association between the consumption of fish and shellfish and bone health among men and postmenopausal women ≥50 years old in Koreans but not in Americans. Further research is needed to determine the effective intake of fish and shellfish on BMD and the risk of osteoporosis in populations with different genetic backgrounds.

## Figures and Tables

**Table 1:** Baseline characteristics of men and postmenopausal women ≥50 years old in the KNHANES and the NHANES ^1^.

	Men		*p*-Value	Women		*p*-Value
	KNHANES (*N* = 3182)	NHANES (*N* = 1435)		KNHANES (*N* = 3972)	NHANES (*N* = 1223)	
Age (year)	60.94 ± 0.20	60.50 ± 0.23	0.171	63.54 ± 0.20	62.59 ± 0.28	0.008
BMI (kg/m^2^)	23.82 ± 0.07	28.40 ± 0.21	<0.001	24.22 ± 0.06	27.97 ± 0.20	<0.001
Alcohol use, *n* (%)	2371 (78.0)	1134 (82.8)	<0.001	1736 (45.8)	671 (63.2)	<0.001
Smoking state, *n* (%)	1014 (35.5)	280 (29.8)	0.019	169 (4.7)	169 (31.8)	<0.001
Regular exercise, *n* (%)	1582 (51.2)	295 (23.0)	<0.001	1467 (37.2)	141 (13.3)	<0.001
Bone mineral density (mg/cm^2^)						
Total femur	932.43 ± 2.84	1007.68 ± 5.68	<0.001	771.66 ± 2.48	843.18 ± 4.80	<0.001
Femoral neck	754.94 ± 2.57	819.10 ± 4.20	<0.001	621.41 ± 2.24	721.84 ± 3.59	<0.001
Lumbar spine	945.62 ± 3.30	1061.88 ± 6.68	<0.001	799.04 ± 2.76	940.97 ± 5.55	<0.001
Dietary intake						
Fish and shellfish (times/month)	23.90 ± 0.40	5.07 ± 0.20	<0.001	22.01 ± 0.41	5.29 ± 0.24	<0.001
Protein (g/day)	74.96 ± 0.86	90.33 ± 1.70	<0.001	51.81 ± 0.57	66.42 ± 0.82	<0.001
Sodium (mg/day)	5573.56 ± 80.20	3781.15 ± 76.47	<0.001	3806.92 ± 56.98	2834.24 ± 52.11	<0.001
Calcium (mg/day)	559.34 ± 8.46	999.35 ± 23.56	<0.001	420.19 ± 7.77	869.8 ± 21.72	<0.001
Family history, *n* (%)	372 (13.1)	135 (13.2)	0.908	637 (16.0)	179 (20.4)	0.010
Medical history, *n* (%)						
Thyroid disease	48 (1.4)	66 (4.9)	<0.001	297 (7.4)	254 (22.3)	<0.001
Rheumatoid arthritis	45 (1.2)	95 (5.2)	<0.001	221 (5.5)	121 (7.3)	0.054
Osteoporosis	267 (6.9)	88 (5.4)	0.098	568 (21.7)	279 (23.5)	0.323

KNHANES, Korean National Health and Nutrition Examination Survey; NHANES, National Health and Nutrition Examination Survey; BMI, body mass index; regular exercise, more than 90 min per week; family history, parents had osteoporosis or fracture; ^1^ Values are mean ± standard deviation or number of subjects (percentage distribution), as appropriate.

**Table 2:** Correlation coefficients between bone mineral densities of total femur, femoral neck, and lumbar spine and the consumption of fish and shellfish ^1^.

	Men ≥50 Years Old		Postmenopausal Women ≥50 Years Old	
	KNHANES (*N* = 3182)	NHANES (*N* = 1435)	KNHANES (*N* = 3972)	NHANES (*N* = 1223)
Total femur	0.0748 ***	-0.0275	0.1611 ***	0.0754
Femoral neck	0.0768 ***	−0.0002	0.1806 ***	0.0697
Lumbar spine	0.0465 **	-0.0124	0.1630 ***	0.0249

KNHANES, Korean National Health and Nutrition Examination Survey; NHANES, National Health and Nutrition Examination Survey; ^1^Partial correlation coefficient after adjusting for BMI, regular exercise, intake of calcium, smoking state, family history of osteoporosis or fracture, and medical history of rheumatoid arthritis; * *p* < 0.05, ** *p* < 0.01, *** *p* < 0.001.

**Table 3:** Multivariate-adjusted odds ratios and 95% confidence intervals for the risk of osteoporosis according to the consumption of fish and shellfish ^1^.

	Quintiles of Consumption of Fish and Shellfish (Times/Month)					
	Q1	Q2	Q3	Q4	Q5	*p* for Trend ^2^
**Men ≥50 years old**						
**Average intake in KNHANES**	4.2	10.9	19.3	30.4	49.7	
Model 1	1.00	0.74 (0.49–1.12)	0.53 (0.33–0.85)	0.52 (0.32–0.83)	0.25 (0.15–0.42)	<0.001
Model 2	1.00	0.92 (0.58–1.45)	0.68 (0.42–1.12)	0.69 (0.42–1.15)	0.35 (0.20–0.59)	<0.001
Model 3	1.00	0.95 (0.60–1.49)	0.70 (0.42–1.16)	0.71 (0.43–1.18)	0.37 (0.20–0.60)	<0.001
**Average intake in NHANES**	0.0	1.5	3.5	5.8	12.2	
Model 1	1.00	0.51 (0.26–1.01)	0.83 (0.39–1.75)	1.08 (0.50–2.35)	0.53 (0.21–1.35)	0.722
Model 2	1.00	0.61 (0.30–1.21)	1.15 (0.52–2.56)	1.31 (0.55–3.12)	0.68 (0.27–1.73)	0.958
Model 3	1.00	0.43 (0.16–1.16)	0.90 (0.30–2.72)	1.12 (0.43–2.89)	0.57 (0.15–2.12)	0.999
**Postmenopausal women ≥50 years old**						
**Average intake in KNHANES**	3.1	8.5	16.6	28.3	49.3	
Model 1	1.00	0.59 (0.47–0.74)	0.53 (0.41–0.69)	0.41 (0.32–0.52)	0.34 (0.26–0.45)	<0.001
Model 2	1.00	0.63 (0.49–0.80)	0.52 (0.40–0.68)	0.42 (0.32–0.54)	0.35 (0.26–0.47)	<0.001
Model 3	1.00	0.63 (0.49–0.80)	0.55 (0.42–0.71)	0.43 (0.33–0.56)	0.36 (0.28–0.50)	<0.001
**Average intake in NHANES**	0.0	1.5	3.6	6.0	11.9	
Model 1	1.00	0.87 (0.50–1.51)	0.57 (0.32–1.03)	0.69 (0.40–1.17)	0.57 (0.36–0.90)	0.025
Model 2	1.00	0.89 (0.51–1.53)	0.56 (0.31–1.03)	0.69 (0.39–1.21)	0.56 (0.35–0.89)	0.022
Model 3	1.00	0.93 (0.48–1.79)	0.61 (0.34–1.06)	0.71 (0.35–1.45)	0.61 (0.38–1.00)	0.065

KNHANES, Korean National Health and Nutrition Examination Survey; NHANES, National Health and Nutrition Examination Survey; ^1^ Adjusted odds ratios were determined by multiple logistic regression analysis; Model 1: crude; Model 2: adjusted for BMI, regular exercise, and intake of calcium; Model 3: adjusted for BMI, regular exercise, intake of calcium, smoking state, family history of osteoporosis or fracture, and medical history of rheumatoid arthritis; ^2^ Likelihood ratio test was used for detection of trends.
